# Site-specific responses of lowland rice to acidulated and calcined phosphate rock fertilizers in the Center-West region of Burkina Faso

**DOI:** 10.1371/journal.pone.0250240

**Published:** 2021-04-19

**Authors:** Monrawee Fukuda, Dohan M. Soma, Shinya Iwasaki, Satoshi Nakamura, Takashi Kanda, Korodjouma Ouattara, Fujio Nagumo

**Affiliations:** 1 Crop, Livestock, and Environment Division, Japan International Research Center for Agricultural Sciences, Tsukuba, Ibaraki, Japan; 2 Département Gestion des Ressources Naturelles/Systèmes de Production, Institut de l’Environnement et de Recherche Agricole, Ouagadougou, Burkina Faso; University of Delhi, INDIA

## Abstract

Soil phosphorus (P) deficiency is a major constraint of crop production in Sub-Saharan Africa. In particular, in the Sudano-Sahelian zone of Burkina Faso, P is rarely replenished as fertilizer supplies are limited in rural areas and because of the socio-economic situation of farmers. There is however, an abundance of local phosphate rock resource in the country. The development of local inorganic P fertilizers to improve crop production and replace the nutrients removed after harvesting, as well as to promote to sustainable agriculture, is desired. This study evaluated the efficiency of low-grade Burkina Faso phosphate rock (BPR)-based P fertilizers, produced by acidulation and calcination-the major fertilizer processing methods, on lowland rice production and the soil factors influencing their effectiveness. The results showed that the acidulated P fertilizers were as effective as conventional commercial P fertilizers on various soil types, textures, and fertility. Calcined P fertilizers were consistently effective on fine-textured soils with high basic fertility. It was found that fine soil texture and basic fertility of the initial soils were important factors in agronomic efficiency of BPR-based fertilizers and the resilience of rice production to climatic variability. It is recommended that soil type, with respect to soil texture, soil properties, inherent fertility, and water availability, should be considered when using BPR-based fertilizers for rice cultivation.

## Introduction

Phosphorus (P) deficient soils, which are widely distributed in semi-arid areas like Burkina Faso, are derived from acidic parent materials that have low levels of P [1] and they can result in limited rice yields [2–4]. The application of P fertilizers is the most promising method by which to improve soil P and crop production [1, 5]. However, soil P replenishment by small-scale farmers to improve crop production is still inadequate because of the socio-economic situation of farmers [6] and the high cost and lack of availability of imported fertilizers [7]. In 2018, rice production in Burkina Faso was 0.16 MT, with an average yield of 0.95 Mg ha^-1^ [8], which is far below the expected national rice production level of 0.84 MT [9]. There is a large amount of available Burkina Faso phosphate rock (BPR) at the Kodjari deposit, and this provides an attractive option by which to increase P inputs and thus address the low levels of rice production [6, 10]. Furthermore, the utilization of locally produced P fertilizers will help to improve sustainable crop production.

Direct applications of BPR were previously found to be effective for rice cultivation in some agroecological zones of Sub-Saharan Africa [11–13]. However, BPR use is still limited due to its low intrinsic solubility and the variability of its effectiveness for rice in different soil conditions [14]. To increase the solubility of BPR and thus enhance agronomic efficiency, fertilizer processing methods such as acidulation by sulfuric or phosphoric acid [10, 15] or calcination using additives [16] have been proposed. However, variations in the agronomic efficiency of P fertilizers result from disparities in their solubility and availability in different soils, and this is controlled by several factors, such as climate, location, soil properties, water conditions, and plant species [5, 15, 17–20]. Effective soil depths and textures are documented as the principal soil parameters governing the availability of water and P in the soil [21].

Previous studies have tried to elucidate the solubility characteristics and factors that impact the effectiveness of partially acidulated phosphate rock-PR (PAPR) and calcined PR. It is known that the P component in PAPR dissolves faster than the original PR [22]. PAPR was more effective than PR in sandy soils when the rainfall > 600 mm [23]. However, the agronomic efficiency of PAPR prepared from unreactive PR seems less predictable [24]. Calcined Brazilian PR (Juquia and Sapucaia) was 66–72% as effective as triple superphosphate (TSP) for paddy rice on clay loam soil, whereas a reactive Gafsa PR (Tunisia) was ineffective [17]. Calcined low-grade Christmas Island PR (Australia) showed a comparable effect to superphosphate (SSP) for radish yields on silt loam soils [25], while there was no significant effect on wheat production [19]. A modified calcination method for low-grade BPR was developed and achieved a high 2% citric acid solubility [26]. These calcined PRs were comparable with TSP at improving rice biomass production in pot experiments [26]. However, the effects of PAPR and calcined PR on rice cultivation in different soil conditions remain poorly understood, especially in West Africa.

Along a soil toposequence in the Center-West region of Burkina Faso, there was a positive relationship between sorghum yields and soil types that had deep effective soil depths and were consequently likely to have higher water-holding capacities when compared to soils with shallow effective depths [27]. It is known that a topography influences soil characteristics and water availability. Moreover, the availability of soil P vary within small distances due to the topography [18, 27]. Thus, the interactions between the site-specific conditions and P fertilization effects require further investigation.

Our study objectives were to evaluate the effectiveness of BPR-based fertilizers, produced by the acidulation and calcination methods, on lowland rice production in different soil conditions in the Sudano-Sahelian climatic zone of Burkina Faso and to reveal the factors controlling P fertilization effects at site-specific level.

## Materials and methods

### Site description

**Fig 1:**
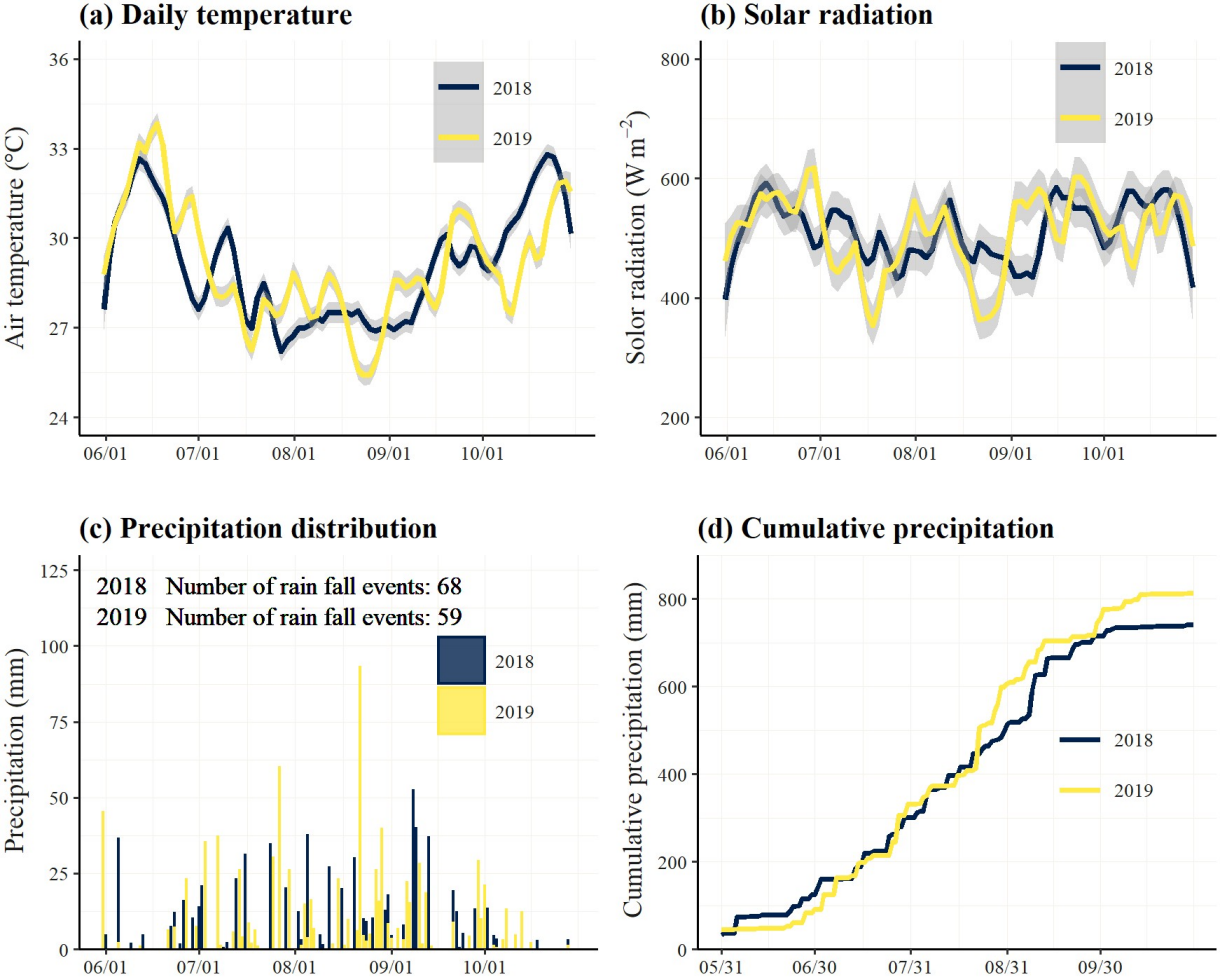
Meteorological data in cropping seasons 2018 and 2019. (a) Daily temperature, (b) Solar radiation, (c) Precipitation distribution, and (d) Cumulative precipitation.

A field experiment was carried out in 2018 and 2019 in the Center-West region of Burkina Faso, which lies in a semi-arid Sudano-Sahelian climatic zone [28]. The rainfall pattern is unimodal with hot and dry seasons from November to April and rainy and humid seasons from May/June to September/October. Annual rainfall is 800 mm on average, and the annual mean temperature is 28 °C. Daily temperature, solar radiation, precipitation distribution, and cumulative precipitation in the 2018 and 2019 cropping seasons are presented in Fig 1.

Cumulative precipitation was 741 mm and 813 mm, and there were 68 and 59 rainfall events, in 2018 and 2019, respectively. There were fewer precipitation events in 2019 but those that did occur were of a higher intensity when compared to those in 2018. Moreover, the cumulative precipitation level during the early stage of plant growth (~June 1 to July 10) in 2019 was lower than in 2018.

Four farmland sites; two in Nassoulou village (namely NAS-D and NAS-M), one in Poa village (namely Poa), and one in Ramongo village (namely Ramongo) villages, located between 12°12′–12°21′N and 2°06′–2°13′W were selected. The sites had different soil types and textures, and degrees of fertility. Soils were classified as Gleyic Lixisols in Poa, Gleyic Ferric Lixisols in NAS-D and NAS-M, and Pisoplinthic Plinthosols in Ramongo. The initial soil physicochemical properties are shown in Table 1.

**Table 1:** Physicochemical properties of the soil samples from the four study sites in the Center-West region of Burkina Faso.

**Site**	**n**	**Sand**	**Silt**	**Clay**	**Total carbon**	**Total nitrogen**	**C/N ratio**	**pH**	
								**H**_**2**_**O**	**KCl**
		**(%)**			**(g C kg**^**-1**^**)**	**(g N kg**^**-1**^**)**			
**NAS-D**	32	52.5 ± 8.8	18.8 ± 3.4	28.8 ± 5.9	7.13 ± 0.97	0.55 ± 0.05	13.0 ± 0.95	6.00 ± 0.31	4.40 ± 0.36
**NAS-M**	32	63.2 ± 5.9	14.9 ± 1.8	21.9 ± 4.8	5.94 ± 0.79	0.52 ± 0.05	11.4 ± 0.73	5.86 ± 0.32	4.36 ± 0.39
**Poa**	40	75.2 ± 3.0	11.9 ± 1.5	12.8 ± 2.5	4.71 ± 0.74	0.43 ± 0.06	10.9 ± 0.54	6.09 ± 0.17	4.71 ± 0.30
**Ramongo**	40	77.8 ± 2.5	11.8 ± 1.8	10.4 ± 1.4	2.87 ± 0.4	0.31 ± 0.03	9.09 ± 0.61	5.13 ± 0.16	4.09 ± 0.05
**Site**	**n**	**EC**	**Bray-1 P**	**Exchangeable cations**				**Base saturation (BS)**	**CEC**
				**K**	**Ca**	**Mg**	**Al**		
		**(S m**^**-1**^**)**	**(mg P kg**^**-1**^**)**	**(cmol**_**c**_ **kg**^**-1**^**)**				**(%)**	**(cmol**_**c**_ **kg**^**-1**^**)**
**NAS-D**	32	2.34 ± 0.52	0.68 ± 0.32	0.09 ± 0.02	5.69 ± 1.52	2.47 ± 0.75	0.22 ± 0.26	72.7 ± 8.24	11.4 ± 2.47
**NAS-M**	32	2.74 ± 1.33	2.10 ± 1.02	0.11 ± 0.05	3.65 ± 2.11	1.15 ± 0.50	0.34 ± 0.22	65.0 ± 12.9	7.31 ± 2.16
**Poa**	40	2.23 ± 0.93	2.62 ± 0.63	0.07 ± 0.03	2.11 ± 0.59	0.42 ± 0.12	0.07 ± 0.06	71.2 ± 14.3	3.73 ± 0.90
**Ramongo**	40	2.12 ± 0.69	3.55 ± 1.29	0.04 ± 0.01	0.31 ± 0.08	0.07 ± 0.02	0.67 ± 0.14	19.3 ± 6.18	2.55 ± 0.93

Values are mean ± SE, EC, Electrical conductivity; P, Phosphorus; K, Potassium; Ca, Calcium; Mg, Magnesium; Al, Aluminum; CEC, Cation exchange capacity.

The field studies did not involve endangered or protected species. The landowners gave permission to conduct the study on these sites.

The soil texture was classified [29] as a sandy loam (SL) in Ramongo and Poa, and sandy clay loam (SCL) in NAS-D and NAS-M. From the soil analysis, NAS-D had the highest clay content followed by NAS-M, Poa, and Ramongo, respectively (Table 1), and the soils with higher clay contents also had higher total carbon (TC) and total nitrogen (TN) contents, and cation exchange capacity (CEC).

Field observations showed that these sites also differed in that some had natural water basins that could potentially contribute water to the adjacent arable areas and micro-topography. There were natural water basins close to the given sites in NAS-D and Poa. Farmers at NAS-D could bring up groundwater by digging shallow wells during dry season vegetable cultivation. Despite having the same Lixisols soil type, NAS-D and NAS-M were located on different elevations, as NAS-M was in the middle, while NAS-D was at the bottom of a flat slope. In Ramongo, the soils were visibly susceptible to drought and substantially drier at the end of the rainy season due to their coarse-textured soil characteristics and the lack of natural water basins nearby.

### Experimental design and field procedures

#### Sources of P fertilizer

The P fertilizers used in this study were prepared using locally available ground BPR from the Kodjari deposit. Two types of calcined BPR (CB) were produced. Briefly, BPR was well mixed with potassium carbonate (K_2_CO_3_) to produce CBk [26] and K_2_CO_3_ + calcium carbonate (CaCO_3_) to produce CBkca, and these were then calcined for 10 minutes at 900 °C for CBk and 1,000 °C for CBkca to increase the P solubility.

Two types of partially acidulated BPR (PAPR), 75% acidulated (PAPR75) and 100% acidulated (PAPR100), were produced by mixing the BPR with 60% sulfuric acid (H_2_SO_4_) [30]. Rates of the acid addition were decided after determining the mineral compositions of the BPR. The CBs and PAPRs were ground into powder before use. In this study, TSP and SSP were used as the positive controls for the CBs and PAPRs, respectively. Some selected properties and the P solubility of the fertilizers were analyzed [31] and are shown in Table 2.

**Table 2:** Selected properties and P solubility of the fertilizers.

Solubility	Unit	BPR	CBk	CBkca	TSP	PAPR75	PAPR100	SSP
**Water solubility (WP)**	% of TP	0.2	2.4	0.0	28.1	28.9	45	91.6
**Alkaline ammonium citrate solubility (SP)**	% of TP	2.5	36.8	45.4	93.6	56.9	83.9	96.8
**2% citric acid solubility (CP)**	% of TP	31.1	74.2	96.2	100	67.3	83.9	96.8
**Total P (TP)**	g P_2_O_5_ kg^−1^	26.6	15.5	20.1	45	21.1	18	19.1
**pH**_**(H2O)**_		7.4	12.3	12.3	3	2.8	2.7	3

BPR, Burkina Faso phosphate rock; calcined BPR (CBs as CBk and CBkca); partially acidulated BPR (PAPRs as PAPR75 and PAPR100); TSP, triple superphosphate; SSP, single superphosphate.

The PAPRs varied in water solubility (WP) from 29% to 45%, alkaline ammonium citrate solubility (SP) varied from 57% to 84%, and the 2% citric acid solubility (CP) varied from 67% to 84%, when expressed as a percentage of the total P (TP) with a pH of 2.7 to 2.8. The CBs contained 0% to 2.4% WP, 37% to 45% SP, and 74% to 96% CP as a percentage of TP with a pH of 12.3. PAPRs had higher WP and SP but lower CP than CB. The WP is the fraction of the P fertilizers that is most rapidly soluble and available for the plants. Other basic extractable-SP and acidic extractable-CP fractions represent the more slowly released P fractions. Some water-insoluble or partially WP fertilizers that vary in SP may be effective as WP fertilizers in certain conditions [5].

#### Fertilizer treatments

Eight fertilizer treatments: 1) control without P application (CT), 2) BPR, 3) CBkca, 4) CBk, 5) PAPR75, 6) PAPR100, 7) TSP, and 8) SSP, were used in a randomized complete block design (RCBD) in 4 m × 4 m plots with four replications in NAS-M and NAS-D, and 2.5 m × 2.5 m plots with five replications in Poa and Ramongo. The sizes of the experimental plots differed between the sites because the farmland space was limited. The rate of the P application was 35 kg phosphorus pentoxide (P_2_O_5_) ha^-1^ (corresponded to 15.3 kg P ha^−1^). All treatments received basal fertilizers of 74 kg N ha^-1^ as urea and 40 kg potassium oxide (K_2_O) ha^-1^ as potassium chloride (KCl). The field trials were carried out for 2 successive years in 2018 and 2019 using the same fields and fertilizer treatments.

#### Rice variety and cultivation

An improved rice (*Oryza sativa* L.) variety FKR19, which is widely used in irrigated rice schemes, was directly seeded using approximately five seeds per hill in 20 cm × 20 cm planting spaces and grown in bunded rice plots under natural rainfed conditions. Rice grains and straw were harvested from designated areas that were 3 m × 3 m (for NAS-M and NAS-D) and 1.6 m × 1.6 m (for Poa and Ramongo) by excluding the 0.5 m rims of the plot.

### Sample collection and analysis

#### Soil samples

Surface soil samples (0–20 cm) were collected from five points per plot to represent the whole treatment before and after cultivation in 2018 and before cultivation in 2019. Composite soil samples were well mixed, air-dried, ground, and passed through a 2-mm sieve. A mixture of soil and distilled water at a ratio of 1:5 was utilized to measure soil pH using a LAQUA pH/ION F-72 (Horiba, Japan) and electrical conductivity (EC) using a COND meter ES-51 (Horiba, Japan). The soil pH_KCl_ was measured in a mixture of soil and 1 M KCl at a 1:5 ratio using LAQUA pH/ION F-72 (Horiba, Japan). Total carbon (TC) and total nitrogen (TN) were determined using dry combustion methods with a Sumigraph NC-220F (Sumika Chemical Analysis Service, Ltd., Japan). Available P was extracted using a Bray-1 extracting solution [32]. The concentrations of P in the filtrate were determined using a colorimetric method [33] with a UV-1800 spectrophotometer (Shimadzu, Japan). Exchangeable bases were extracted using a 1.0 M ammonium acetate solution (pH 7.0). Then, the cation concentrations were determined using inductively coupled plasma atomic emission spectrophotometry (ICP-AES) with an ICPE-9000 (Shimadzu, Japan). To determine the CEC, the residues after the exchangeable base extractions were then washed with 80% ethanol. Then, the saturated ammonium (NH_4_^+^) was extracted four times with 1 mol L^-1^ KCl (pH 7.0) and determined using the salicylate method [34], with the continuous flow analysis of using the Auto Analyzer III (BL-TEC K. K., Tokyo, Japan). Base saturation (%) was calculated as the sum of exchangeable cations divided by CEC × 100.

#### Plant samples

Three-hill samples per plot for the rice plants grown outside the designated area for grain yield investigation were collected to represent the whole sampling plot 4, 8, 12, and 16 weeks after sowing (WAS) and used for straw P concentration analysis. Rice straw samples were oven-dried at 70 °C and finely ground. Samples were dry-ashed with a muffle furnace at 550 °C for 2 hours and dissolved with 0.5 M HCl. The concentration of P in the extract was determined using a colorimetric method [33] with a UV-1800 spectrophotometer (Shimadzu, Japan). The P concentrations in the rice straw in the 2018 cropping season were determined only, due to unavailability of plant samples in the 2019 cropping season.

### Calculation

Biomass was calculated using the sum of the grain and straw yields. The harvest index (HI) was calculated [35] as follows

$$HI\;(\%)=\frac{Grain\;yield}{Biomass}\;x\;100$$

Agronomic efficiency of the P fertilizer (AEP, kg grain kg^-1^ P) or an increased grain yield per kg P applied was calculated [36] as follows

$$AEP\;(kg/kg)=\frac{\left(Grain\;in\;fertilized\;treatment-Grain\;without\;fertilization\right)}{Fertilizer\;P\;applied}$$

### Statistical analysis

Statistical analysis was performed using R version 4.0.0 [37]. The effect of the year, site, treatment, and their interactions were analyzed using three-way analysis of variance (ANOVA). Multiple comparisons using Shaffer’s Modified Sequentially Rejective Bonferroni Procedure [38] were conducted if a significant difference was detected. The effect size of the source was evaluated using the eta squared (η2). Principal component analysis (PCA) was used to depict the general relationships between the yield components and the soil characteristics and the seasonal differences in yield. A 2-year data set was subjected to PCA after standardization to compare the variables with different scales and units. Variables included grain yield (GY); biomass, harvest index (HI); soil characteristics included total carbon (TC), total nitrogen (TN); soil acidity (pH_KCl_, extractable Al); available Bray-1 P (AP); cation exchange capacity (CEC); base saturation (BS); and soil particles (sand, silt, clay), respectively.

## Results

### Grain yield, biomass, and harvest index (HI)

The effects of the P fertilizers in 2018 and 2019 on the rice grain yields were obtained from all study sites (S1 Table). The average grain yield, biomass, and HI for the years, sites, and fertilizer treatments are presented in Table 3. Across the years, sites, and P fertilizer treatments, the preliminary analysis revealed that the biomass and HI correlated well with the grain yield (S2 Table). The three-way ANOVA showed the effects of the year (Y), site (S), and treatment (T), and their interactions with grain yield, biomass, and HI (Table 4). The results indicated that the grain yield and biomass were significantly affected by Y, S, and T, probably representing the effects of the seasonal climate (mainly precipitation), site characteristics (e.g., soil types, soil property, water condition), and fertilizer solubility. The HI was significantly affected by Y and S, indicating that it was mainly influenced by seasonal climate and site characteristics but not by fertilizer solubility.

**Table 3:** Average grain yield, biomass, and harvest index (HI) for the years, sites, and fertilizer treatments.

Year	Grain yield	Biomass	Harvest index	Site	Grain yield	Biomass	Harvest index	Treatment	Grain yield	Biomass	Harvest index
	(Mg ha^-1^)		(%)		(Mg ha^-1^)		(%)		(Mg ha^-1^)		(%)
**2018**	4.14 ± 1.03 b	7.18 ± 1.82 b	58.0 ± 8.2 b	**Ramongo**	2.45 ± 1.10 a	4.86 ± 1.70 a	48.9 ± 8.7 a	**CT**	2.90 ± 1.26 a	5.09 ± 1.77 a	54.2 ± 9.8
**2019**	3.23 ± 2.22 a	6.32 ± 2.22 a	50.1 ± 8.6 a	**NAS-D**	3.83 ± 1.17 b	7.90 ± 2.34 c	48.4 ± 6.3 a	**BPR**	3.31 ± 1.12 ab	6.16 ± 1.52 b	53.0 ± 10.0
				**NAS-M**	4.21 ± 0.83 c	7.59 ± 1.58 c	55.9 ± 4.6 b	**CBkca**	3.40 ± 1.33 b	6.26 ± 1.99 b	53.1 ± 10.5
				**Poa**	4.37 ± 0.90 c	7.05 ± 1.04 b	62.2 ± 8.2 c	**CBk**	3.76 ± 1.18 bc	6.86 ± 2.01 bc	54.0 ± 8.8
								**PAPR100**	3.98 ± 1.16 c	7.17 ± 1.80 cd	55.2 ± 8.3
								**PAPR75**	4.00 ± 1.00 c	7.32 ± 1.73 cd	54.7 ± 8.2
								**TSP**	4.05 ± 1.23 c	7.42 ± 2.10 d	54.5 ± 9.9
								**SSP**	4.08 ± 1.31 c	7.67 ± 2.44 d	53.6 ± 9.4

Mean values with different letters in the same column are significantly different at *P* < 0.05. CT, control or without P fertilizer; BPR, Burkina Faso phosphate rock; calcined BPR (CBs as CBk and CBkca); partially acidulated BPR (PAPRs as PAPR75 and PAPR100); TSP, triple superphosphate; SSP, single superphosphate.

**Table 4:** The interaction effects of the years (Y), sites (S), and fertilizer treatments (T) on the grain yield, biomass, and harvest index.

Grain yield					Biomass					Harvest index				
Source	df	F-ratio	*P*-value	η^2^	Source	df	F-ratio	*P*-value	η^2^	Source	df	F-ratio	*P*-value	η^2^
**Y**	1	106.9	***	0.12	**Y**	1	38.5	***	0.04	**Y**	1	163.4	***	0.16
**S**	3	114.3	***	0.40	**S**	3	104.7	***	0.33	**S**	3	135.9	***	0.40
**T**	4	13.5	***	0.11	**T**	4	21.8	***	0.16	**T**	4	0.8	0.598	0.01
Y * S	3	9.3	***	0.03	Y * S	3	26.8	***	0.09	Y * S	3	51.8	***	0.15
Y * T	4	0.3	0.963	0.00	Y * T	4	0.7	0.630	0.01	Y * T	4	1.4	0.208	0.01
S * T	12	2.2	**	0.05	S * T	12	5.1	***	0.11	S * T	12	1.2	0.248	0.02
Y * S * T	12	1.1	0.320	0.03	Y * S * T	12	1.2	0.279	0.03	Y * S * T	12	1.8	*	0.04

*, **, *** indicate significant difference at *P* < 0.05, 0.01, and 0.001, respectively.

**Fig 2:**
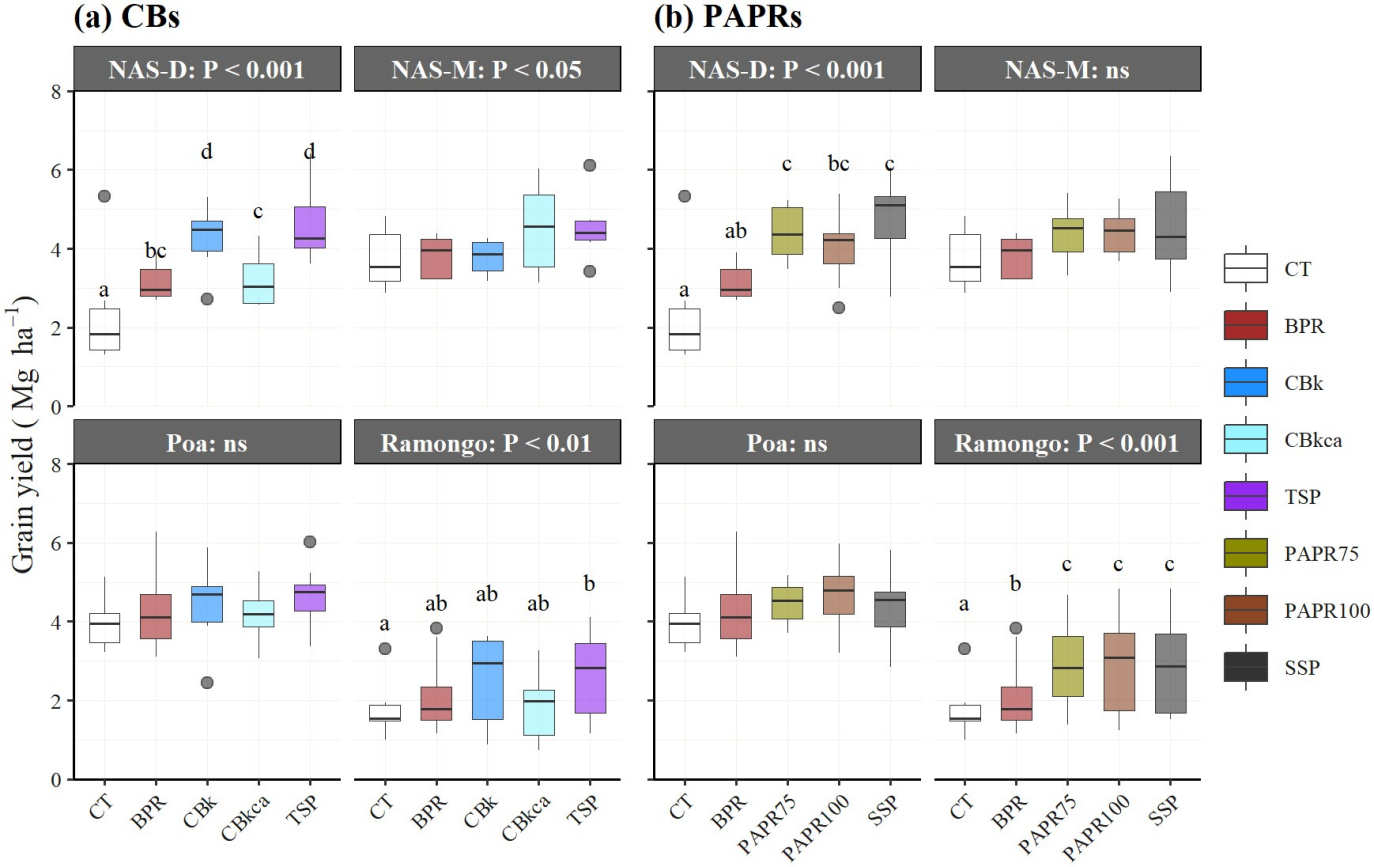
Effects of the P fertilizers on the rice grain yields at each study site. CT, Control or without P fertilizer; BPR, Burkina Faso phosphate rock; calcined BPR (CBs as CBk and CBkca); partially acidulated BPR (PAPRs as PAPR75 and PAPR100); TSP, triple superphosphate; SSP, single superphosphate.

Across the sites and treatments, grain yield, biomass, and HI were significantly higher in 2018 than in 2019 (Table 3). A significant interaction effect of Y and S indicated that the seasonal differences in yield production depended on the sites (Table 4). Across the years and sites, P fertilization increased grain yield by up to 41% with the SSP and 40% with the TSP when compared with the control (CT). BPR-based fertilizers, PAPR100, PAPR75, and CBk increased the yield by up to 38%, 37%, and 30%, while CBkca and BPR increased it by 17% and 14%, respectively (Table 3). There were no interaction effects of Y and T on grain yield, implying that the P fertilizers performed consistently in both years (Table 4). However, the interactions of S and T implied that there were variations of the P fertilization effects at the different sites. Therefore, the effects of the P fertilizers on the rice yields at each study site were further investigated using combined grain yields for 2018 and 2019 (Fig 2).

A significant difference in grain yields among the treatments was observed at NAS-D and Ramongo but not at Poa or NAS-M. The effects of the P fertilization were as follows, in descending order: TSP = CBk > CBkca = BPR > CT and SSP = PAPR75 ≥ PAPR100 ≥ BPR ≥ CT, respectively at NAS-D, and TSP ≥ CBk = CBkca = BPR ≥ CT and SSP = PAPR100 = PAPR75 > BPR > CT, respectively at Ramongo. PAPRs showed comparable performances with SSP and had significantly higher grain yields in NAS-D than those in Ramongo. While, CBk was comparable to TSP in NAS-D. The performance of the CBs was comparable with TSP in Ramongo, although there was no significant difference with the CT.

### Principal component analysis (PCA) of the important soil characteristics for rice yields

**Fig 3:**
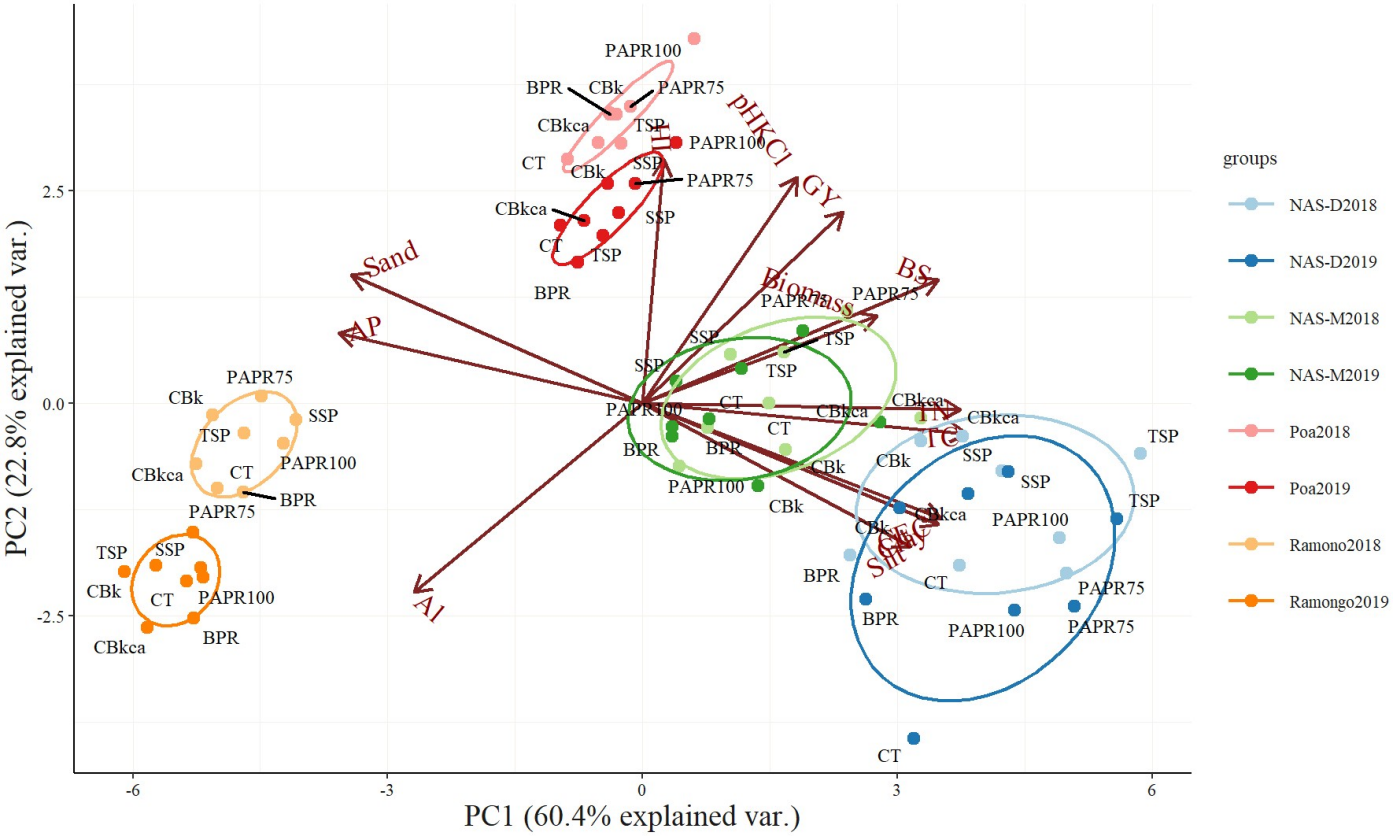
Principal component analysis (PCA) of the soil characteristics that are important for rice yield at the study sites. Variables included grain yield (GY); biomass; harvest index (HI); soil characteristics [total carbon (TC), total nitrogen (TN)]; soil acidity (pH_KCl_, extractable Al); available Bray-1 P (AP); cation exchange capacity (CEC); base saturation (BS); and soil particles (sand, silt, clay).

A PCA was performed to visualize the effects of the soil factors that explain the variability of the P fertilization effects between the sites and years (Fig 3).

The 13 variables were converted to a set of two linearly uncorrelated principal components (PCs) that contributed to 83.2% of the total variance. The components of the PCA and the contribution rates of the variables are shown in S3 Table. PC1 explained 60.4% of the total variance reflecting the contributions of the fine particles (clay and silt) and TC, TN, CEC, and BS on the biomass and grain yield following P fertilization. This finding may suggest that the high basic soil fertility in fine-textured soil affected the solubilization and the efficiency of P fertilizers, resulting in increased grain and biomass production. PC2 explained 22.8% of the total variance, mainly reflecting the correlation of HI and grain yield, and soil acidity (pH_KCl_ and Al), suggesting that the changes in soil acidity contributed to the changes in grain yield and HI, particularly in 2019 when seasonal differences clearly appeared for PC2. Moreover, the seasonal differences were most substantial in Ramongo, followed by Poa, where the variables in PC1 were lower than at other sites (Fig 3). This suggests that the changes in the coarse-textured soil acidity in 2019, which were affected by the climatic conditions, may influence the grain yield and HI.

### Straw P concentration

**Fig 4:**
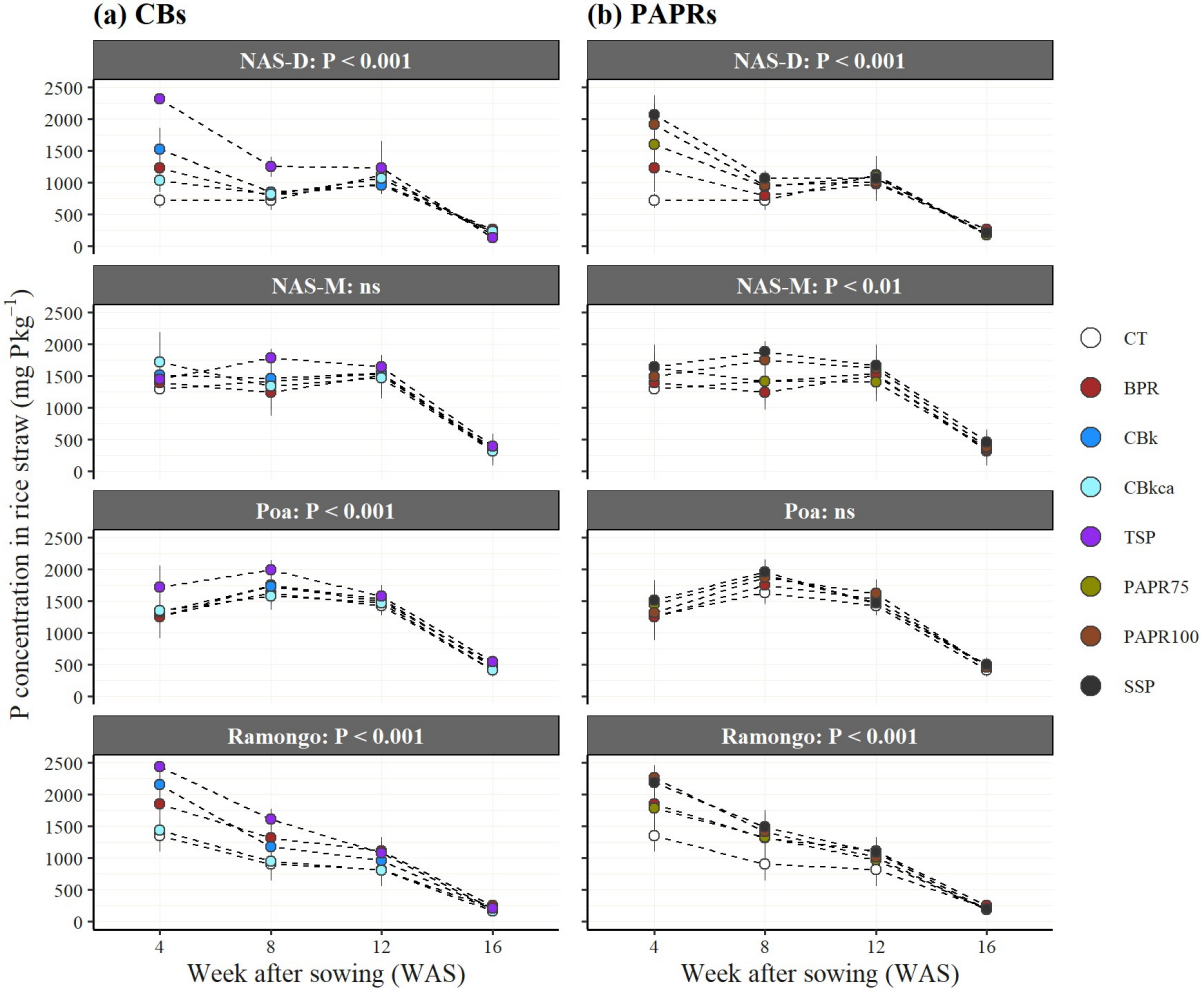
Phosphorus concentrations in the rice straw 4, 8, 12, and 16 weeks after sowing. CT, control without P fertilizer; BPR, Burkina Faso phosphate rock; calcined BPR (CBs as CBk and CBkca); partially acidulated BPR (PAPRs as PAPR75 and PAPR100); TSP, triple superphosphate; SSP, single superphosphate. Bars indicate standard deviation. *** indicates a significant difference at *P* < 0.001 and ns is not significant.

The P concentration in the rice straw, which was affected by the P fertilization, significantly differed between sites, treatments, and growing periods in both PAPRs and CBs (Fig 4). The differences in the P concentrations between the treatments was notable in 4 WAS, and significant differences were observed in NAS-D and Ramongo. The P concentrations were highest in 4 WAS and gradually decreased with time.

**Fig 5:**
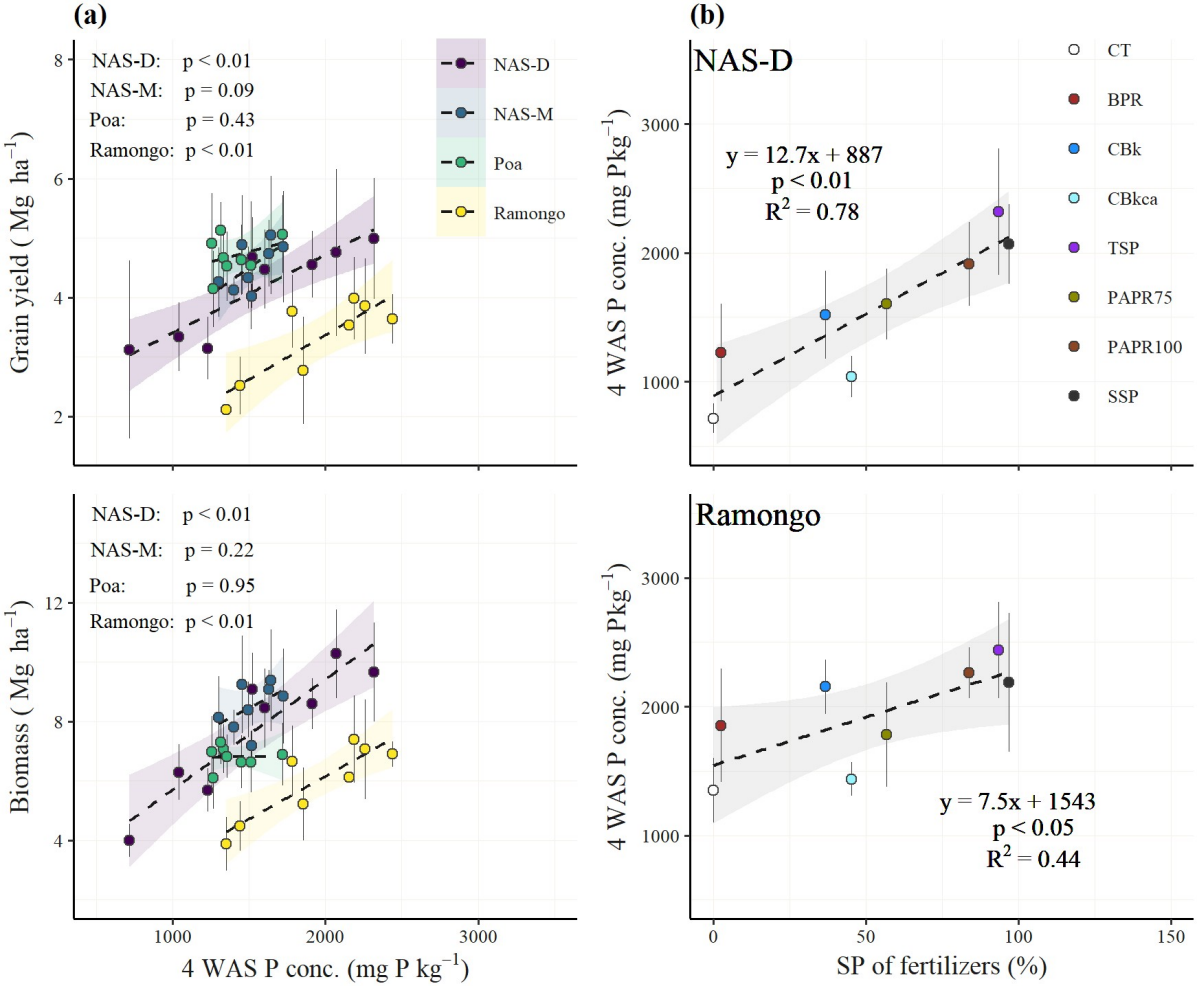
Relationships between the P concentrations of the straw and the yield and the fertilizer’s alkaline ammonium citrate solubility (SP). (a) Relationships between the straw P concentrations 4 weeks after sowing (WAS) and the grain and biomass yields. (b) Relationships between the alkaline ammonium citrate solubility of the P fertilizers (SP) and the 4 WAS straw P concentrations in NAS-D and Ramongo. Error bars are standard deviation (*n* = 4 in NAS-D and NAS-M, *n* = 5 in Poa and Ramongo). The colored areas surrounding the regression lines represent 95% confidence interval. CT, control without P fertilizer; BPR, Burkina Faso phosphate rock; calcined BPR (CBs as CBk and CBkca) and partially acidulated BPR (PAPRs as PAPR75 and PAPR100); TSP, triple superphosphate; SSP, single superphosphate.

The relationships of the 4 WAS P concentrations with the biomass and grain yields are shown in Fig 5a. In NAS-D and Ramongo, where the grain yield was significantly responsive across the treatments, grain yield and biomass were positively correlated with the 4 WAS P concentrations with a high coefficient of determination (R^2^; 0.79 and 0.65 in NAS-D and Ramongo, respectively for grain yield, and 0.82 and 0.73 in NAS-D and Ramongo, respectively for biomass). In NAS-D and Ramongo, the 4 WAS P concentration was significantly higher due to the increasing alkaline ammonium citrate solubility (SP) of the P fertilizers (Fig 5b). The levels of SP were highest in the SSP and TSP, followed by PAPRs, CBs, and BPR, respectively (Table 2).

### Agronomic efficiency of the P fertilizer (AEP)

**Fig 6:**
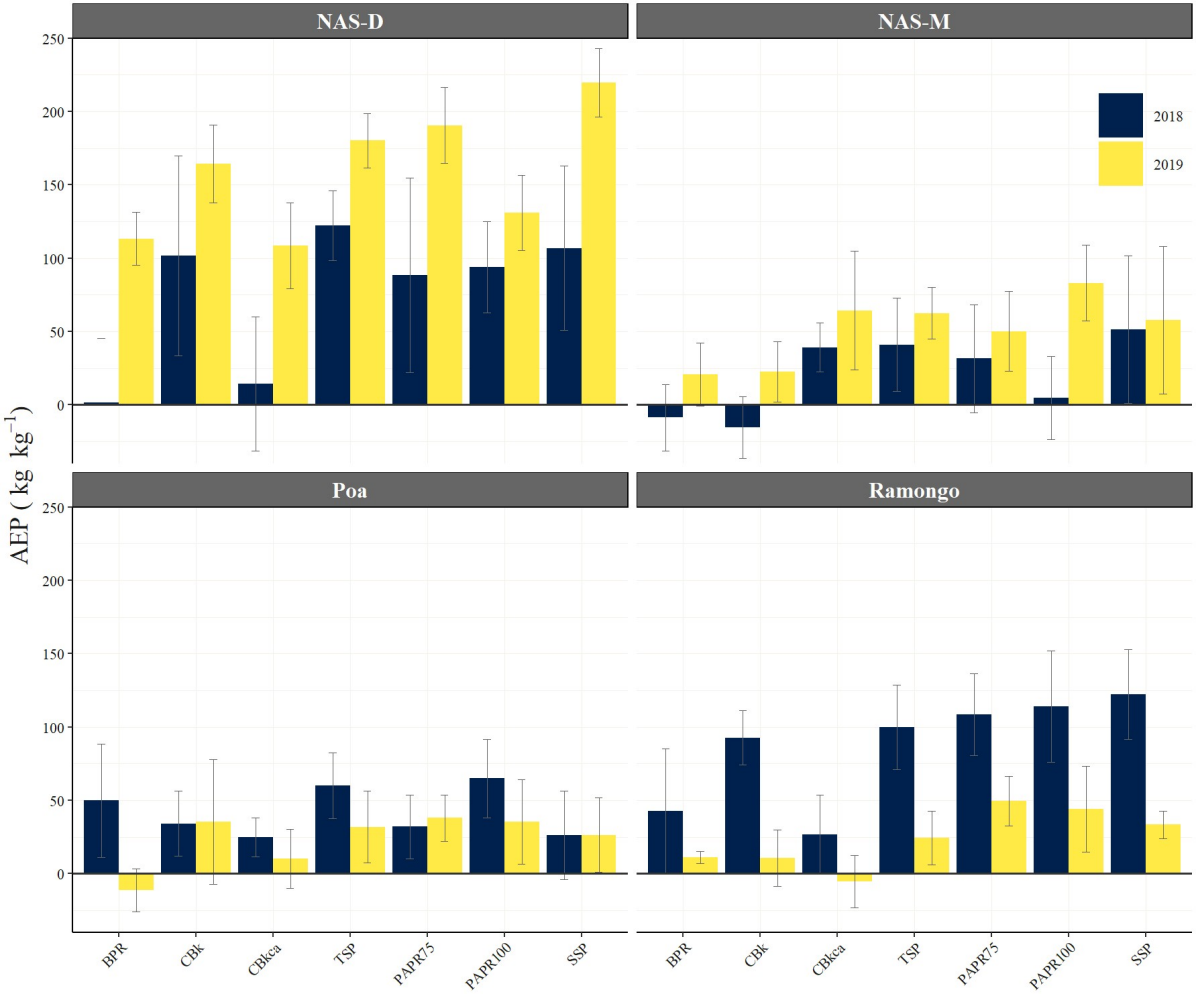
Agronomic efficiency of the P fertilizers (AEP) obtained in 2018 and 2019. Error bars are standard error (*n* = 4 in NAS-D and NAS-M; *n* = 5 in Ramongo and Poa). BPR, Burkina Faso phosphate rock; calcined BPR (CBs as CBk and CBkca); partially acidulated BPR (PAPRs as PAPR75 and PAPR100); TSP, triple superphosphate; SSP, single superphosphate.

The AEP was evaluated as shown in Fig 6 and was found to vary among the sites and years. Focusing on the sites (i.e., Ramongo and NAS-D) where the grain yield was significantly different between the treatments, the AEP in NAS-D were found to be equal and higher than that of Ramongo in 2018 and 2019, respectively. The two sites showed an opposite trend in their AEPs for annual variations. In the NAS-D, the AEP was higher in 2019 than in 2018 for all treatments. On the other hand, in Ramongo, the AEP was higher in 2018 than in 2019, and the reduced AEP was more apparent in the CBs than the PAPRs.

The relationship between the AEP and initial soil physicochemical properties in 2018 and 2019 across the four study sites was analyzed using Pearson’s correlation coefficient (Table 5). In 2018, the AEP had no significant correlation with any of the soil properties, while there were significant correlations in 2019 with many soil properties.

**Table 5:** Pearson’s correlation coefficient (*r*) for the AEP and soil properties.

Soil properties		2018			2019		
		n	*r*	*P*-value	n	*r*	*P*-value
**Sand**	(%)	126	0.00	0.98	126	-0.59	***
**Silt**	(%)	126	0.07	0.44	126	0.55	***
**Clay**	(%)	126	-0.03	0.77	126	0.58	***
**Total carbon**	(g C kg^-1^)	126	-0.13	0.14	126	0.55	***
**Total nitrogen**	(g N kg^-1^)	126	-0.19	0.03	126	0.46	***
**CEC**	(cmolc kg^-1^)	126	-0.07	0.45	126	0.62	***
**Available P**	(mg P kg^-1^)	126	0.11	0.23	126	-0.46	***
**pH**_**KCl**_		126	-0.03	0.78	126	-0.01	0.90
**Exchangeable Al**	(cmolc kg^-1^)	126	-0.01	0.88	126	-0.17	0.06
**Base saturation**	(%)	126	-0.16	0.08	126	0.33	***

*** indicates significant difference at *P <* 0.001.

### Changes in soil pH and available P

The soil pH and available P (Bray-1) are important factors when considering P fertilization. The changes in soil pH and available P of the initial soils were compared across the years, sites, treatments (Tables 6 and 7). Overall, soil pH decreased, while available P increased after the first cropping season. However, the alteration of soil pH and available P differed according to the soil properties and types of fertilizer.

**Table 6:** Soil pH across the sites, and its changes due to the different fertilization treatments before rice cultivation in 2018 and 2019.

Year	Soil pH _(H2O)_	Treatment	Changes in pH _(H2O)_				
	All sites		All sites	NAS-D	NAS-M	Poa	Ramongo
**2018**	5.75 ± 0.46b	**CT**	0.01 ± 0.22	-0.01 ± 0.10	-0.06 ± 0.35	-0.13 ± 0.12	0.21 ± 0.13
**2019**	5.47 ± 0.37a	**BPR**	0.03 ± 0.32	-0.26 ± 0.30	0.23 ± 0.33	-0.14 ± 0.12	0.28 ± 0.15
		**CBk**	0.10 ± 0.31	-0.13 ± 0.16	-0.01 ± 0.13	-0.05 ± 0.12	0.53 ± 0.17
		**CBkca**	0.03 ± 0.23	-0.12 ± 0.15	0.00 ± 0.11	-0.13 ± 0.16	0.32 ± 0.11
		**TSP**	0.01 ± 0.28	-0.26 ± 0.13	0.18 ± 0.16	-0.17 ± 0.22	0.27 ± 0.16
		**PAPR75**	-0.07 ± 0.30	-0.36 ± 0.12	0.04 ± 0.22	-0.22 ± 0.29	0.23 ± 0.10
		**PAPR100**	-0.05 ± 0.26	-0.28 ± 0.28	-0.07 ± 0.20	-0.12 ± 0.18	0.24 ± 0.08
		**SSP**	-0.14 ± 0.27	-0.39 ± 0.32	-0.14 ± 0.28	-0.13 ± 0.12	0.06 ± 0.23
		**All treatments**	-0.05 ± 1.15	-0.23 ± 0.22	0.02 ± 0.24	-0.14 ± 0.17	0.27 ± 0.18

Mean (± standard error) values with different in the same column are significantly different at *P* < 0.05.

**Table 7:** Soil available P across the sites, and its changes due to the different fertilization treatments before rice cultivation in 2018 and 2019.

Year	Available P (mg P kg^-1^)	Treatment	Changes in available P (mg P kg^-1^)				
	All sites		All sites	NAS-D	NAS-M	Poa	Ramongo
2018	2.33 ± 1.37a	**CT**	-0.36 ± 0.59	-0.06 ± 0.08	0.12 ± 0.55	-0.41 ± 0.70	-0.94 ± 0.20
2019	3.11 ± 1.31b	**BPR**	-0.50 ± 1.18	0.29 ± 0.24	-0.93 ± 2.30	-0.29 ± 0.58	-1.01 ± 0.63
		**CBk**	-0.19 ± 2.05	-0.02 ± 0.19	0.80 ± 0.46	0.68 ± 0.82	-1.98 ± 3.33
		**CBkca**	-0.18 ± 0.79	0.15 ± 0.20	0.41 ± 0.43	0.12 ± 0.20	-1.22 ± 0.71
		**TSP**	0.46 ± 1.05	0.54 ± 1.55	0.21 ± 0.52	1.10 ± 1.05	-0.04 ± 0.83
		**PAPR75**	-0.04 ± 0.84	-0.01 ± 0.39	0.26 ± 0.86	0.60 ± 0.57	-0.94 ± 0.65
		**PAPR100**	-0.14 ± 0.79	0.16 ± 0.13	-0.15 ± 1.02	-0.18 ± 1.18	-0.32 ± 0.59
		**SSP**	0.58 ± 1.00	0.34 ± 0.44	0.31 ± 0.33	0.77 ± 0.20	0.79 ± 1.94
		**All treatments**	-0.05 ± 1.15	0.17 ± 0.57	0.13 ± 1.01	0.30 ± 0.86	-0.71 ± 0.18

Mean (± standard error) values with different letters in the same column are significantly different at *P* < 0.05.

## Discussion

### Yield and yield responses at the study sites

Overall, the grain yields were significantly different between the sites (Table 3). According to the PCA (Fig 3), biomass and grain yield production were explained well by the soil characteristics (PC1), i.e., fine (silt and clay) particles and basic soil fertility (TC, TN, BS, and CEC). The grain yield at Ramongo was significantly lower than the other sites (Table 3) due to the low soil fertility (Table 1), shallow effective soil depth, and low water-holding capacity of its coarse-textured Plinthosols [27]. The quick disappearance of water due to the low water retention could result in insufficient levels of available water for rice growth throughout the season. Furthermore, acidic soils (pH < 5.5) can limit P-availability to plants via soil fixation with the Al, Fe, or Ca cations and the toxicity of Al and manganese [39]. Moreover, soils containing low base saturation (BS) or basic cations such as K^+^, Ca^2+^, and Mg^2+^ and a low CEC could adversely affect grain and biomass yields.

Grain and biomass yields and the HI were significantly different between the years and sites, with respect to their interacting effects (Table 4). The lower grain yields in 2019 were associated with the decreased HI and biomass and were exaggerated in the coarse-textured soils (i.e., Ramongo and Poa; Fig 3). The reduction in the grain yields was relatively small in fine-textured soils (i.e., NAS-D and NAS-M; S1 Table, Fig 3), emphasizing the vulnerability of the coarse-textured Plinthosols to the changes in the rainfall distribution patterns (Fig 1). Rice might suffer from fluctuating soil water inputs when there are high precipitation levels, but the durations of such events are short. A shortage of water or water stress in the field was reported to significantly decrease the HI [40], resulting in decreased grain yields. Likewise, plant biomass production is also linearly coupled with the amount of water transpired [41]. Furthermore, intense precipitation may cause excessive drainage and leaching of the base-cations, lowering the soil pH [39] and imposing unfavorable soil conditions, which, in turn, hinders the HI and rice production. Changes in the soil acidity had significant impacts on rice production and the efficiency of BPR-based fertilizers in the coarse-textured soils in Ramongo and Poa (Fig 3).

In this study, the Lixisols (i.e., soils in NAS-D, NAS-M, and Poa) generally had higher clay contents and CEC when compared to the Plinthosols (i.e., soils in Ramongo; Table 1). Furthermore, Lixisols in the Center-West region of Burkina Faso were found to have a deeper effective soil depth and higher water-holding capacity in comparison to the Plinthosols [27]. Clays are essential for the stabilization of organic matter [42, 43]. Significant levels of TC and the CEC representing the soil organic matter can increase the exchange sites available to retain nutrients [44, 45], water availability, and air quality for root growth and the rooting environment [46]. In addition, they can also improve the soil physical properties and protect against soil erosion [42], possibly ameliorating the seasonal effects on the efficacy of the P fertilizers. This may explain the higher rice grain yield in both seasons and the smaller reduction in yield obtained from these sites (S1 Table, Table 3) although the impacts of seasonal climate variability were expected in 2019.

### Site-specific effects on P fertilization

In Poa and NAS-M, grain yields were consistently high (Fig 2), ranging from 3.20 to 4.26 Mg ha^-1^ without P fertilizer application (S1 Table) and the rice did not significantly respond to the P fertilization regardless of the types of fertilizer (Fig 2), indicating that P was not limiting for rice grain yields at these sites. This was contradicted by the low P status in the soils (less than 4 mg P kg^-1^) at all sites (Table 1). The soils may already have sufficient P levels for yield production resulting in an insignificant response to the additional P fertilizers [47]. However, the soils in Ramongo and NAS-D appeared to have significant responses to the P fertilizers (Fig 2), suggesting that some other factors were governing the efficiency of the P fertilizers.

In Poa, the soils had a high base saturation and soil pH but low exchangeable Al^3+^ (Table 1), resulting in satisfactory grain yields (Table 3). Furthermore, a natural water basin near the experimental field may add available water and nutrients to the cultivated field, ensuring that there would be no water deficit for the plants throughout the cropping season. At the NAS-M site, the basic soil fertility was higher than it was at Poa and Ramongo (Table 1, Fig 3). NAS-M is in the middle of a flat slope and is slightly higher in elevation than NAS-D. This difference in position on the slope was assumed to affect the availability of water and nutrients [18], and consequently P fertilization. However, the yield obtained from NAS-M was not limited by P fertilization, soil fertility, or water conditions. A hypothesis was that the extended aerobic soil conditions during the cropping season might induce organic matter decomposition and the release of essential nutrients into the soils. It is well documented that organic matter decomposition is faster in aerobic than flooded soils [48] and the nutrient turnover rates of organic matter in the Sudano-Sahelian zone with high soil temperatures and microfauna were rapid [42]. However, the reason for obtaining high grain yields from this P-deficit soil even with no P fertilization, were not fully understood in this investigation.

The P fertilization effects were significant in Ramongo and NAS-D. However, the PAPRs and CBs showed a different trend (Fig 2). The PAPRs (PAPR100 and PAPR75) and SSP were effective on both the P-deficient fine-textured Lixisols (NAS-D) and the coarse-textured Plinthosols (Ramongo). The CBs showed a comparable effect to the TSP in Ramongo, although there was no significant difference with respect to the CT. The CBk was as effective as TSP, and more effective than the CBkca in NAS-D, which may be due to a higher WP (Table 2). It is possible that the fine-textured Lixisols in the NAS-D provided adequate water to dissolve the CBs. Moreover, higher C and CEC would bind more cations (K^+^, Ca^2+^, and Mg^2+^) or lime resources [49] and provide a sink for the fertilizer-dissolved products [23], which would in turn facilitate the continuous solubilization of CBs. Simultaneously, high water availability also results in advantageous conditions that facilitate root expansion and affect P-uptake efficiency in lowland soils [50] as well as the accessibility of nutrients [48], and may this result in CBs being profoundly effective.

Principally, acidulation and calcination increased the P solubility (WP, SP, and CP) of BPR (Table 2). The WP and SP are commonly considered to be available P for plants [5] and are guaranteed on the fertilizer labels [51]. The CP is used as an index for effective P in chemical fertilizers. Fertilizers containing high CP levels are considered to have slow-release P [52]. The P fertilizers’ solubility is the most important factor influencing the initial and residual fertilization effects [5]. This study has confirmed the statistically significant increase of rice biomass and grain yield, particularly in two high P responsive sites at Ramongo and NAS-D (Fig 5), because of the increasing SP in the BPR-based fertilizers and their positive linear relationship with increasing straw P concentrations during the early vegetative phase (4 WAS). Plant P concentrations are known to facilitate early plant vegetative establishment and increase the quantity of remobilized-C from vegetative biomass to grain yield [50, 53]. Moreover, an adequate P supply for rice plants is critical at early stages as plants often uptake half of their seasonal P requirements by the time they accumulate a quarter of their total seasonal dry matter [5, 35]. These results showed the vital importance of the increased SP in BPR-based fertilizers after applying fertilizer processing methods to improve the P solubility of the low grade BPR.

Single applications of any of the different fertilizer types to the lowland rice fields resulted in the cultivated soil acidity being slightly increased (Table 6). Soil pH alterations were varied and inconsistent because of several natural processes causing the release of protons (H^+^) into the soils [54]. Further investigation on the effects of BPR-based fertilizers on soil acidity are needed. Although, the levels of soil available P were widely varied among the different types of fertilizers and their sites, the overall initial soil P was increased from 2.33 mg P kg^-1^ in 2018 to 3.11 mg P kg^-1^ in 2019 (Table 7). This implied that applied fertilizers were able to replenish the soil P status. Continuous P fertilization should be encouraged to correct P deficiencies in soils and maintain crop productivity.

### Agronomic efficiency of P fertilizers (AEP)

The AEP or increased yield per unit of P applied represents the direct production impacts of an applied fertilizer [55], and is often used to evaluate optimum P management practices [36]. The AEP values of PAPRs were comparable to TSP and SSP in both NAS-D and Ramongo, and CBs showed a consistent performance in the NAS-D because of the reasons mentioned above (Fig 6). These results suggested that the PAPRs had a considerable direct impact on production and could be used to substitute the high-costs of imported fertilizers on both Plinthosols and Lixisols, which were widely distributed in this region. The results also suggested that at the sites with fine-textured soils, both PAPRs and CBs were useful. The PAPR100 provides higher level of available P than PAPR75 due to the higher degree of acidulation [10, 15]. However, this study has proved that PAPR75 and PAPR100 had similar fertilization effects, suggesting that PAPR75 was preferable because it had a lower degree of acidulation, resulting in much lower cost of production and higher economic advantage. Currently, all fertilizer manufacturing processes consume large amounts of energy [56]. Country with low energy costs or that use more renewable energies could have lower production costs, and making fertilizer manufacturing processes more affordable. Furthermore, this would also increase the competitive ability of locally-producible P fertilizers.

The AEPs of the P fertilizers at the NAS-D site increased in 2019 compared with 2018 in all treatments, including the directly-applied BPR (Fig 6). This may be attributed to the sufficiency of the cumulative precipitation in 2019 (Fig 1) and the large water retention capacity of the fine-textured Lixisols. On the other hand, the AEPs were drastically reduced in the coarse-textured Plinthosols of the Ramongo site (Fig 6) as they were affected by the erratic rainfall distributions in 2019 (Fig 1). This phenomenon was particularly notable for the CBs, suggesting that the agronomic efficiency of PAPRs on rice yield production tended to have more climatic resilience than the CBs and BPRs. As soil dries, the available P is reduced [57], its solubility in the soil declines sharply [58], and deficiencies become more significant [59]. This effect, in turn, enhances the requirements for high solubility P applications [4]. Furthermore, Table 5 clearly showed that the agronomic efficiency of BPR-based fertilizers in 2019 was improved and dependent on the initial soil properties before rice cultivation, particularly, being fine-textured (i.e., clay and silt) and having basic fertility (i.e., CEC, TC, TN, and BS) which involve the improvement of soil physical properties, stability of soil aggregate, water holding capacity [42], and the supply of an adequate soil water and protons (H^+^) to spontaneously solubilize P fertilizers [23]. These results showed that fine soil textures and basic fertility were important factors to improve the agronomic efficiency of BPR-based fertilizers and the resilience of rice production to climatic variability. These factors should thus be considered when using BPR-based fertilizers to achieve satisfactory agronomic efficiency levels for rice cultivation.

The recent annual rainfall was enough for lowland rice cultivation in the region, however the climatic variability in rainfall distributions, both temporal and spatial, resulting from the shorter and more unpredictable rainy seasons over the previous three decades in Burkina Faso has increased [60]. The erratic rainfall distributions within a cropping season in combination with the soil physicochemical properties of a specific site and the solubility of fertilizer should be integrated into nutrient management planning and implementation.

The influences of the actual soil water conditions on the effectiveness of the BPR-based fertilizers, the optimal BPR-based fertilizer application rates on lowland rice environments, and the costs and benefits of the fertilizers used in this region have not yet been investigated and should be addressed in future projects.

## Conclusion

Lowland rice responded variably to BPR-based P fertilizers mainly due to the solubility of the fertilizer, soil characteristics, climatic conditions, and their complex interactions. Rice grain yield was generally higher in the Lixisols than the Plinthosols soil types because of beneficial advantages of the fine soil texture and inherent fertility on P fertilization effects. For P fertilizer highly responsive sites, PAPRs were as effective as the imported conventional fertilizers regardless of soil type, soil texture (i.e., fine and coarse), or climatic conditions (i.e., irregular rainfall distribution), due to the high levels of P solubility. The CBs were consistently effective on fine-textured Lixisols because of the continuous solubilization of the CBs facilitated by the high-water availability and basic fertility. Alkaline ammonium citrate solubility P in BPR-based fertilizers increased P concentrations in the rice straw at the early vegetative phase, which contributed to an increase in grain and biomass production. The initial presence of fine soil particles (i.e., clay and silt) and basic fertility (i.e., CEC, TC, TN, BS) in soils played an important role in improving the agronomic efficiency of the fertilizers and the resilience of rice production to climatic variability by hindering yield loss when the soil water levels fluctuated. Soil type considerations, with respect to soil texture, soil properties, inherent fertility, and water availability, are thus required when using BPR-based fertilizers for rice cultivation in the Center-West region of Burkina Faso.

## Supporting information

S1 Table Rice grain yields (Mg ha^-1^) with the different P fertilizer treatments. CT, Control or without P fertilizer; BPR, Burkina Faso phosphate rock; calcined BPR (CBs as CBk and CBkca); partially acidulated BPR (PAPRs as PAPR75 and PAPR100); TSP, triple superphosphate; SSP, single superphosphate.
(XLSX)

S2 Table Pearson’s correlation matrix between grain yield, biomass, and harvest index. *r* is Pearson’s correlation coefficient, **, *** indicate significant difference at *P* < 0.01, and < 0.001, respectively.
(XLSX)

S3 Table Components for PCA and contribution rates of the original variables. (XLSX)
